# Two types of cleavage, from zygote to three cells, result in different clinical outcomes and should be treated differently

**DOI:** 10.3389/fcell.2024.1398684

**Published:** 2024-06-03

**Authors:** Luba Nemerovsky, Yehudith Ghetler, Amir Wiser, Mattan Levi

**Affiliations:** ^1^ IVF Unit, Department of Obstetrics and Gynecology, Meir Medical Center, Kefar Sava, Israel; ^2^ Faculty of Medicine, Tel Aviv University, Tel Aviv, Israel

**Keywords:** zygote, direct cleavage, Z-score, embryo, blastocyst

## Abstract

**Research Question:**

What is the utilization rate of embryos that exert inadequate zygote cleavage into three daughter cells?

**Design:**

This study used a retrospective dataset from a single IVF Unit. A total of 3,060 embryos from 1,811 fresh IVF cycles were analyzed. The cleavage pattern, morphokinetics, and outcome were recorded. Only 2pn embryos, fertilized by ejaculated sperm, and cultured in a time-lapse system for at least 5 days were included. We generated three study groups according to the embryo’s cleavage pattern: (I) Control, normal cleavage (*n* = 551); (II) fast cleavage, zygote to three cells within 5 h (*n* = 1,587); and (III) instant direct tripolar cleavage (IDC) from zygote to three cells (*n* = 922).

**Results:**

The rate of usable fast cleavage blastocysts was 108/1,587 (6.81%) and usable control blastocysts was 180/551 (32.67%). The time of PN fading and from fading to first cleavage differed significantly between the three groups. Although the pregnancy rate of control and fast cleavage blastocysts were comparable (40.35% and 42.55%, respectively), the amount of instant direct cleavage embryos that reached blastocyst stage was neglectable (only four embryos out of 922 analyzed IDC embryos) and unsuitable for statistical comparison of pregnancy rates.

**Conclusion:**

Our results indicate the need to culture instant direct cleavage embryos for 5 days, up to the blastocyst stage, and avoid transfer of embryos that are fated to arrest even when their morphological grade on day 3 is acceptable, whereas fast cleavage embryos could be transferred on day 3 when there is no alternative.

## Introduction

Identifying embryos with the highest implantation potential and early identification of those that are abnormal is very challenging in ART (Anagnostopoulou et al., 2022; Coticchio et al., 2022). Time-lapse imaging in incubating systems enable continuous observation of embryos’ morphological and kinetic parameters. Zygote morphology is one of the earliest embryonic stages investigated. Several zygote grading systems have been proposed (Nicoli et al., 2013) based on the size and distribution of pronuclei (PN) and nucleolus precursor bodies (NPB). Evaluating the size of the PN just before their membranes breakdown is an effective indicator of the embryo’s potential to result in a live birth (Otsuki et al., 2017). NPB are aggregations of dense fibrillar material that are visible in the PN of fertilized oocytes. Spatial distribution and size of these particles is associated with chromatin rearrangement during fertilization, in preparation for the first cleavage (Cavazza et al., 2021), as they serve as the major heterochromatin-organizing sites. The arrangement of NPB in human zygotes at the pronuclear interface is an indicator of efficient chromosome clustering (required for correct unification of the parental genomes after fertilization) and suggests accurate chromosome segregation and adequate embryo development (Scott, 2003; Cavazza et al., 2021). Chromosome segregation errors, such as misaligned, unattached, or lagging chromosomes, occur mainly in the first cleavage cycle (Currie et al., 2022).

Time-lapse cinematography reveals the occurrence of abnormal cleavage patterns, such as abnormal zygote mitosis resulting in three or more daughter cells, that are otherwise morphologically indistinguishable (Milewski and Ajduk, 2017). Mitotic abnormalities that arise in early cleavage stages affect a large portion of the lineage of the embryonic cells and have a critical effect on the developing embryo (Currie et al., 2022). By using time-lapse monitoring, we can observe cleavage of one cell into three daughter cells through either single tripolar mitosis or two consecutive mitoses separated by a very short interval (cleavage in less than 5 h), defined as direct cleavage (DC; Rubio et al., 2012). This phenomenon might occur at the first, second, or even later cleavage cycles. The mechanisms causing multipolar spindles are not clear, involving abnormal centriole distribution, abnormal centriole replication, or additional formation of a microtubule organizing center, leading to an additional spindle (Kalatova et al., 2015; Ottolini et al., 2017). DC in early stages strongly correlates with impaired blastocyst formation, implantation, and clinical outcome, whereas DC at later stages has a milder impact (Zhan et al., 2016). In two-pronuclear (2PN) human embryos, the frequency of DC at the first cleavage varied widely, ranging from 8.3% to 26% (Athayde Wirka et al., 2014; Yang et al., 2015; Zhan et al., 2016). Moreover, the exact timing of DC could assist in predicting the future of the developing embryo (Bamford et al., 2022). The aim of this study was to differentiate between zygote instant direct cleavage (IDC), defined as cleavage directly into three daughter cells, and fast cleavage (FC), defined as cleavage within less than 5 h from zygote to three daughter cells, comparing and investigating their occurrence and clinical outcomes. We also analyzed zygote morphological parameters of IDC embryos and compared them with those of normally cleaving siblings, in search of signs to predict and identify abnormal cleavage patterns at the zygote stage.

## Materials and methods

### Research approval

This retrospective cohort study was approved by the institutional Helsinki committee, No. 0043-22-MMC from 09/02/2022.

### Study design and participants

The study was conducted from 2014 to 2022. A total of 6024 ART cycles were performed during this period. Only 2PN embryos, fertilized by ejaculated sperm and cultured in time-lapse incubators (EmbryoScope1, Vitrolife, Sweden) for more than 113 h, were retrospectively analyzed. This timeframe was selected as minimal period from insemination (both ICSI and IVF) to the occurrence of the fifth day of incubation in our laboratory protocol (the latest insemination was at 14:00 of day 0 and the earliest annotation was at 7:00 of day 5). In our unit, the EmbryoScope image acquisition system is set to capture images from seven focal planes for each embryo every 15 min during the entire culture period. The time-lapse video created from these images allowed monitoring of specific cell cycle events, as well as embryo morphology. A total of 3,060 embryos from 1,811 cycles were included. The time-lapse annotations were performed independently by three senior embryologists following the same annotation protocol. A rigorous IQC program is used in our laboratory, with an average of CV = 6.2 in the past 3 years.

### Embryo culture and study groups

**FIGURE 1:**
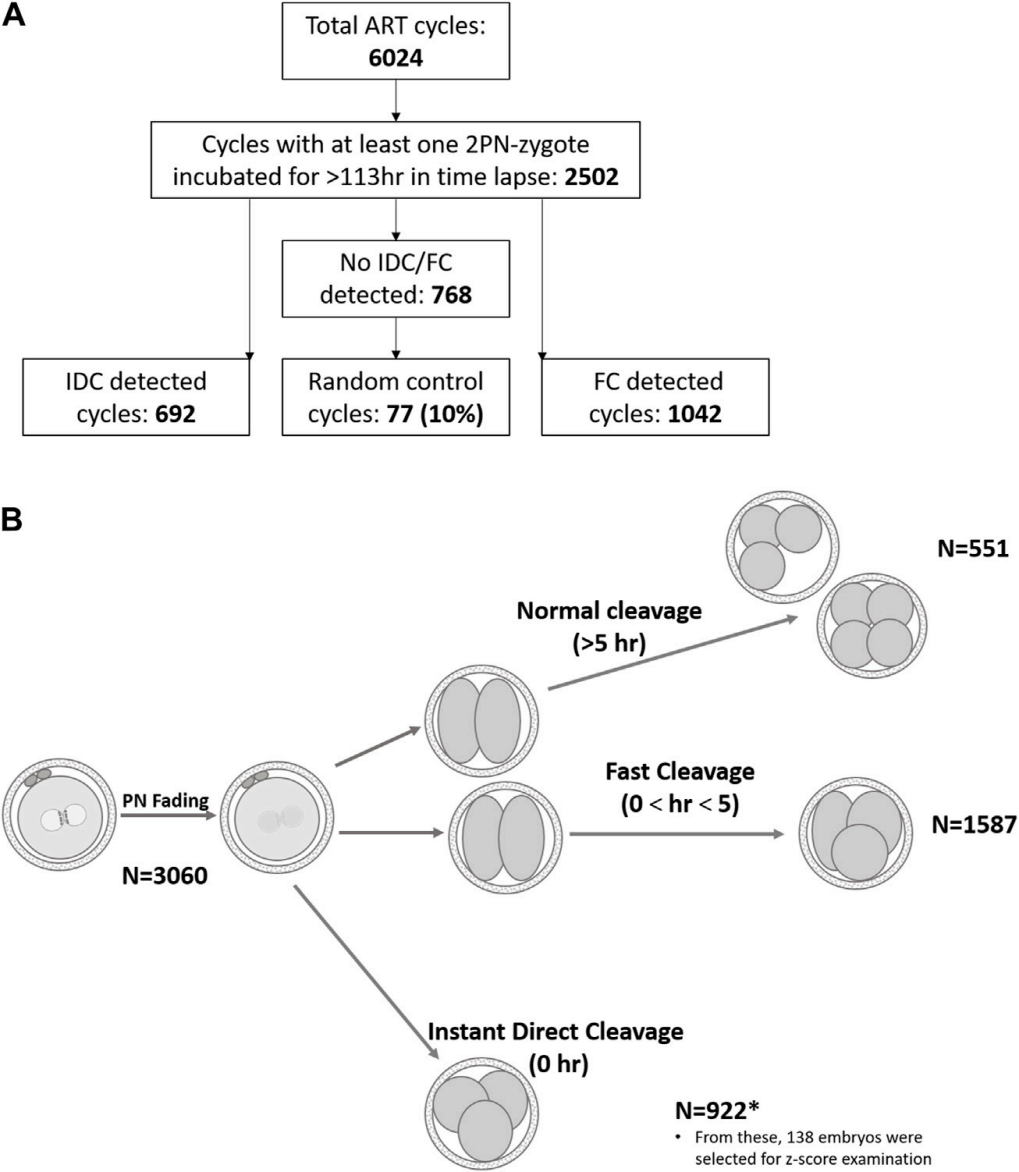
Schematic demonstration of retrospective data analyzed. **(A)**. Treatment cycles subgrouping. **(B)**. Graphical illustration of cleavage patterns and their prevalence. Normal cleavage was defined as cc2 > 5 h (Wong et al., 2010) and fast cleavage as cc2 < 5 h (Rubio et al., 2012). The length of the second cell cycle, cc2 = t3–t2.

All embryos in our laboratory are cultured in single-step Global culture media, (LifeGlobal, Brussels, Belgium) with incubation conditions of 37°C, 5% (±0.3) O_2_, and 5.5% (±0.5) CO_2_. Day 5 embryo grading was performed according to Gardner’s classification (Gardner and Schoolcraft, 1999). According to unit policy, blastocysts BB or above were used, whereas embryos classified as C were not transferred or cryopreserved. In this study, first cleavage patterns were classified as follows (Figure 1): instant direct cleavage (IDC) from zygote to three or four daughter cells, fast cleavage (FC) from zygote to three or four daughter cells in less than 5 h, and normally cleaving embryos were defined as three to four cells in more than 5 h (Wong et al., 2010). Follow-up of these embryos and their outcomes were monitored: arrested (developmental stage recorded), blastocyst used (transferred or cryopreserved), and blastocyst discarded (classified as C grade of ICM or TE). Interval from fertilization to PN fading and from PN fading to first cleavage was also recorded.

### Zygote morphological assessment

Meticulous assessment in search of predictive signs for upcoming IDC was performed in a series of consecutive treatment cycles. The measures of Z-score and classifications were performed by a single senior embryologist. A total of 138 cycles out of 692 IDC cycles analyzed met the following inclusion criteria: IDC–from zygote to three or four daughter cells (FC within less than 5 h was excluded), two visible pronuclei without major overlapping in the zygote, at least one sibling neighbor 2PN embryo with normal cleavage to two cells and analyzable pronuclei, and time-lapse culture up to day 5/6.

#### Measures

For every cycle meeting the inclusion criteria, the IDC embryo, as well as the sibling neighbor 2PN embryo with normal cleavage to two cells, which served as own control to avoid bias, were analyzed as follows: last image was taken just before nuclear envelope breakdown (Zygote grading based on Scott, 2003, pronuclei diameter measured, number, size, and distribution of the NPB were classified); interval from PN fading to first cleavage was measured; embryonic stage was monitored up to day 5/6; the fate of each embryo was documented; the number of “usable” blastocysts (for ET or cryopreservation) and arrested or rejected blastocysts as well as the last developmental stage were also documented. An arrested embryo was defined as an embryo that did not reach the blastocyst stage, remaining in earlier cleavage stages up to day 5 of culture. A rejected embryo was defined as an embryo that continued to cleave to the blastocyst stage, but the resulting blastocyst was of poor quality (less than 3BB according to Gardner’s classification) and therefore unsuitable for use (transfer or cryopreservation).

### Statistical analysis

All analyses were performed using SPSS 23.0 (IBM Corp., Armonk, NY, United States). Chi-square test was used to compare rates and proportions. Normality of the data was assessed using Kolmogorov-Smirnov test. ANOVA was used to analyze continuous variables. If normal distribution was not detected, and/or homogeneity of distribution was not met, the Kruskal-Wallis test was performed and was followed by the original FDR method of Benjamini and Hochberg *post hoc* analysis. Student’s t-test was applied for Z-score analysis of two experimental groups. All *p*-values were tested as two-tailed and considered significant if <0.05.

## Results

Our results show that, out of 14,462 embryos that were 2PN and incubated for >113 h, 82.7% (11,953/14,462) resulted in normal cleavage and 17.3% were direct cleavage (922/14,462 IDC and 1,587/14,462 FC). A total of 922 IDC, 1,587 FC, and 551 control embryos, resulting from 692, 1,042, and 77 treatment cycles, respectively, were included in this study. Patient’s age, number of aspirated oocytes, and insemination method were not significantly different among groups. Significant differences were recorded only for morphokinetic events of PN fading time and time from fading to the first cleavage (Table 1). Among the 922 IDC embryos analyzed, 881 (95.54%) arrested and did not reach the blastocyst stage, as compared to 1,120/1,587 FC embryos (70.57%) and 264/551 (47.91%) controls (Table 1). In the control group, 180 embryos were usable (57 transferred and 123 frozen), whereas only 108 were usable in the FC group (47 transferred and 61 frozen) and only four were usable in the IDC group (two transferred and two frozen). Although 41 IDC embryos formed blastocysts, only four (0.43%) were of usable grade. The proportion of arrested, rejected, and usable blastocysts differed significantly among the groups (Table 1). The outcomes of usable transferred blastocysts originating from FC were comparable to the controls and resulted in similar pregnancy rates (40.35% and 42.55%, respectively). Although a smaller proportion of FC embryos reached blastocyst stage compared to embryos that underwent normal first cycle cleaving (29.43% vs 52.09%, respectively), the pregnancy rate of the transferred blastocysts was comparable to the general pregnancy rate (42.55%). The IDC embryos did not result in any pregnancies (Table 1). The morphological assessment of IDC zygotes *versus* sibling, normally cleaving controls was performed in 138 treatment cycles. Patient age, number of retrieved oocytes, and ICSI proportion did not differ from the data reported in Table 1 (data not shown). Zygote scores (adapted from Scott, 2003) are summarized in Table 2. The IDC embryos arrested significantly earlier than their sibling control embryos in the Z-Score analyzed cycles (3.16 ± 0.66 vs 4.33 ± 0.93 days, respectively; *p* <0.0001). None of the other parameters analyzed, such as PN size and nucleoli number and size, were associated with IDC (Table 2).

**TABLE 1:** Patient data and morphokinetics (average ± SD), Embryo development and outcomes, Outcomes of usable blastocysts.

	Control	FC	IDC	PV
Number of embryos	551	1,587	922	^__^
Number of cycles	77	1,042	692	^__^
Female age (years)	34.69 ± 5.01	33.69 ± 5.92	33.90 ± 6.14	NS
Aspirated oocytes/cycle	11.24 ± 8.24	12.51 ± 7.25	12.36 ± 7.27	NS
% ICSI cycles	68.83	59.88	63.73	NS
PN fading time (hr.)	24.68 ± 4.29	27.19 ± 5.86	29.45 ± 8.62	<0.0001
Time from fading to cleavage (hr.)	2.38 ± 2.12	3.03 ± 2.45	4.20 ± 4.01	<0.0001
Usable blastocysts (%)	180/551 (32.67)	108/1,587 (6.81)	4/922 (0.43)	<0.0001
Rejected blastocysts (%)	107/551 (19.42)	359/1,587 (22.62)	37/922 (4.01)	<0.0001
Arrested embryos (%)	264/551 (47.91)	1,120/1,587 (70.57)	881/922 (95.55)	<0.0001
Pregnancy per transfer (%)	23/57 (40.35)	20/47 (42.55)	0 (0)	NE

PN, Pronuclei; FC, fast cleavage; IDC, Instant direct cleavage; NS, not significant; NE, not eligible for statistical analysis. Usable blastocysts–transferred or frozen embryos.

**TABLE 2:** Z-Score, Pronuclei (PN), and nucleoli measurements of IDC embryos (average ± SD).

	Control	IDC	PV
Z-Score 1 (%)	29.71	38.41	NS
Z-Score 2 (%)	25.36	20.28	NS
Z-Score 3 (%)	41.3	34.06	NS
Z-Score 4 (%)	3.62	7.25	NS
Diameter of PN (µm)	27.25 ± 2.42	27.30 ± 2.22	NS
Delta between PN sizes (µm)	2.47 ± 2.53	2.44 ± 2.1	NS
Nucleoli number	4.60 ± 1.48	4.51 ± 1.49	NS
Nucleoli Asymmetry (%)	29.71	28.99	NS
Day of embryo arrest	4.33 ± 0.93*	3.16 ± 0.66	<0.0001

FC, fast cleavage; IDC, Instant direct cleavage; NS, not significant.

## Discussion

The use of time-lapse incubation revealed several abnormal cleavage patterns (Boucret et al., 2021). Cleavage of one cell into three daughter cells was defined as direct cleavage (DC). This phenomenon might happen during various cleavage cycles. DC was earlier identified as cleavage from one to three cells within 5 h (Rubio et al., 2012). However, the phase of embryonic life at which this phenomenon occurs has a major impact on the outcome. The current study “fine-tuned” the first developmental stages, discriminating between tripolar cleavage from zygote into three daughter cells as IDC and from zygote to three daughter cells within 5 h as FC, as suggested by Ciray et al. (2014) and described as trichotomous *versus* rapid. Our results demonstrate that this differentiation is crucial because the outcomes of these embryos differ. Most IDC embryos arrested at day 3 and no pregnancies were achieved in this group. Due to these small numbers, no statistical analysis was suitable. When the zygote divides directly into three daughter cells, the chromosomal distribution among these cells is chaotic and most cells in the resulting embryo are highly aneuploid (Chatzimeletiou et al., 2005). When the embryo cleaves first into two cells and then one of the blastomeres divides again within 5 h, at least one of the blastomeres in the resulting three-cell embryo is normal. Although presumably mosaic, some of these FC-developing embryos can reach the blastocyst stage and result in pregnancy. Capalbo et al. (2021) reported that low- and medium-grade mosaic embryos have similar reproductive potentials to uniformly euploid embryos. Human embryos exhibit self-correction mechanisms through their ability to eliminate abnormal blastomeres as cell fragments or through apoptosis (Orvieto et al., 2020; Coticchio et al., 2021).

By analyzing the timetable of the first cell cycle, we found significant differences between IDC, FC, and control embryos. PN fading time, as well as time from fading to first cleavage, were significantly longer in both abnormal cleavage patterns as compared to control blastocysts. Significantly longer times to PN fading were reported in poor quality embryos that failed to blastulate (Vera-Rodriguez et al., 2015), as well as in embryos carrying nonviable translocations compared with those carrying potentially viable translocations (Braude et al., 1988). The time between PN fading and first cleavage is of special interest because it can be measured in both conventional IVF and in ICSI. Vera-Rodriguez et al. (2015) reported that the interval between PN fading and first cleavage measured in a time-lapse incubation system was significantly longer in aneuploid-embryos compared to euploid embryos, as confirmed by single-cell RT-qPCR. These kinetic differences might reflect the maternal genome because embryonic genome activation and expression occur around the four-to eight-cell stage (Braude et al., 1988). Abnormal cleavage patterns are characterized by delayed first cleavage and a high proportion of developmental arrest. The proportion of arrested embryos in the current study differed significantly between IDC, FC, and controls. The IDC embryos arrested significantly earlier than their sibling control embryos in the Z-Score analyzed cycles. It is possible that the arrested IDC embryos failed to activate the embryonic genome due to chaotic chromosomal distribution.

In search of early signs of IDC, we analyzed several morphological zygote parameters in detail, as zygote morphology was reported as predictive of treatment outcomes (Nicoli et al., 2013). Moreover, suboptimal PN clustering at the interphase of the zygote might lead to segregation errors (Cavazza et al., 2021). In the current study, we did not find any significant differences concerning Z-scores, PN diameters, nucleoli appearance, or number among IDC embryos and their sibling controls. Therefore, we suggest that the Z-score cannot predict future cleavage patterns. Analyzing the cohort of treatment cycles in which IDC embryos originated revealed that at least one usable blastocyst was available in 93.30%, indicating that the treatment cycle was adequate. In search of tools for effective embryo assessments that can predict reproductive outcomes, several morphokinetic algorithms have been proposed that also consider the early cleavage timetable (Milewski and Ajduk, 2017). Wong et al. (2010) identified a correlation between the ability to achieve the blastocyst stage with an interval from the end of the first division and the onset of the second (the length of the second cell cycle) in human embryos. Divisions that are too fast may result in incorrect segregation of the genetic material and lead to aneuploidy, whereas divisions that are too slow might be a sign of dysfunction in cell cycle checkpoints.

## Conclusion

Based on our results, we propose that the decision regarding the fate of IDC and FC embryos should rely on the pattern of cleavage during the first developmental cycles, since the potential outcomes differ significantly. Most IDC embryos stopped at day 3 developmental stage. The proportion of FC embryos that arrested after day 3 was also significantly greater than that of the controls. Therefore, we recommend not to transfer IDC at day 3, even when their morphological grade is good, but to culture them for 5 days, to the blastocyst stage, instead. On the other hand, FC embryos could be transferred on day 3 when there is no alternative. Further investigation should be conducted regarding the aneuploidy of FC embryos compared to controls.

## Data Availability

The raw data supporting the conclusions of this article will be made available by the authors, without undue reservation.
